# Molecular docking analysis and evaluation of the antibacterial and antioxidant activities of the constituents of *Ocimum cufodontii*

**DOI:** 10.1038/s41598-021-89557-x

**Published:** 2021-05-12

**Authors:** Muhdin Aliye, Aman Dekebo, Hailemichael Tesso, Teshome Abdo, Rajalakshmanan Eswaramoorthy, Yadessa Melaku

**Affiliations:** Department of Applied Chemistry, School of Applied Natural Science, Adama Science and Technology University, P.O.Box 1888, Adama, Ethiopia

**Keywords:** Medicinal chemistry, Chemistry

## Abstract

*Ocimum cufodontii* ((Lanza) A.J.Paton) has been traditionally used in Ethiopia against bacteria*.* The extracts of the leaves and roots of *O. cufodontii* after silica gel column chromatography furnished compounds **1–5**, compounds **3** and **4** are new natural products. The oil from the hydro-distillation of the leaves, after analyzed with GC–MS, has led to the identification of *β*-caryophyllene as a principal component, suggesting the essential oil as medicine and spices to enhance the taste of food. The constituents of *O. cufodontii* were assessed for their antibacterial activity against *E. coli, K. pneumonia, S. typhymurium* and *S. aureus.* The best activity was displayed against *S. aureus* by the hexane extract of the roots, compound **4**, and the essential oil with an inhibition zone of 17, 15, and 19 mm, respectively. Molecular docking analysis revealed that compound **1** has better docking efficiency and forms hydrophobic interactions with five amino acids (ARG192, PHE196, GLU185, GLU193, and LYS189). This suggests that the compounds may act as potential inhibitors of DNA gyrase. The constituents were also assessed for their antioxidant activities using DPPH, ferric thicyanate and ferric reducing power assay. The hexane extracts of the roots inhibited the DPPH radical and peroxide formation by 90.5 and 83%, respectively, suggesting the potential of the extract as an antioxidant. Furthermore, the hexane extract of the roots of *O. cufodontii* exhibited the maximum reducing power compared with the EtOAc and methanol extracts. Hence, the activity displayed herein indicated as the plant has great potential as a remedy for diseases caused by bacteria and radicals.

## Introduction

Since ancient time, plants of the genus *Ocimum* (Lamiaceae) are commercially significant because of their nutritional, aromatic, ornamental, and medicinal importance^1,2^. *Ocimum* has been a key traditional medicine in various parts of the world to treat fevers, stomachache^3^, headaches, coughs, diarrhea and as stimulants and an emetic^2,4^. A lot of research is being done to test the biological effects of some species of the genus *Ocimum*. Scientific sources revealed *Ocimum* has antidiabetic, antipyretic, wound healing, antioxidant, hypolipidemic, bactericidal, and larvicidal activities^4–9^. The genus *Ocimum* is strongly aromatic because of essential oils which comprise monoterpenes, sesquiterpenes, and phenylpropanoids^10^. It is also recognized as a safe and rich source of phenolic antioxidants and flavonoids^11^. The essential oils from plants of this genus are important in pharmaceutical, flavoring, perfumery, fragrance, and cosmetic industries^12,13^.

The genus *Ocimum* comprises over 150 recognized species of herbs and shrubs widely distributed in various parts of the world^14^. *O. cufodontii*, *O. basilicum*, *O. sanctum*, *O. citriodorum,* and *O. gratissimum* are among few *Ocimum* species commonly found in Ethiopia. *O. cufodontii* is a popular hedge plant in Ethiopia, traditionally been used to relieve pain, fever, and inflammation besides its use as antibacterial agent*. O. cufodontii*, locally named in Ethiopia as “*Gororsisa*” in Afan Oromo, is distributed in Borana, Harar, Arsi, and Bale^15^. The plant is also found in East Africa including Somalia and Kenya. Despite the extensive traditional use of this plant against various life-threatening diseases, there is no scientific study on the roots and leaves of *O. cufodontii*. Hence, in view of the attributed medicinal significance of the plant and its availability as an indigenous resource, the present work was undertaken to explore the chemical constituents and some biological activities of the leaves and roots of *O. cufodontii*. Therefore, this paper describes for the first time the molecular docking analysis, antibacterial, and antioxidant activities of the extracts and constituents of the leaves and roots of *O. cufodontii*.

## Materials and methods

### General experimental procedure

Melting points of the isolated compounds were done using melting point apparatus (BUCHI, Germany). Analytical TLC was run on a 0.25 mm thick layer of silica gel GF254 (Merck) on aluminium plate. Detection of spots on TLC was done either by spraying with vanillin or by observation under UV lamp. Purification of compounds was done using column chromatography over silica gel (230–400 mesh). The UV and NMR spectra were measured using UV–Vis spectrophotometer (PG instruments, UK) and Bruker Avance 400 spectrometer operating at 400 MHz, respectively. Mass spectra were run on a JMS HX-110 double focussing mass spectrophotometer, using MeOH as a solvent and glycerol as a matrix on the target. Samples were ionized by bombardment with xenon (gas) atom. Methanol (99.99% GC grade, United Kingdom), *n*-hexane (99% Ranchem industry and trading), Ethyl acetate (98% UNI-CHEM chemical reagents), Ethanol (100% GC grade Mumbai, India) and DPPH (Sigma) were purchased from Addis Ababa (NEWAY PLC, chemicals).

### Plant material collection and identification

The roots and leaves of *O. cufodontii* were collected on October 15, 2017 from Merti, Arsi Zone, Oromia region, Ethiopia. *O. cufodontii* is a wild growing plant collected after getting permission from Merti district administration office, Arsi Zone of Oromia Regional State. The collection of the plant material comply with institutional, national and international guidelines including IUCN Policy. The plant was identified by Mr. Shambel Alemu at the Biology Department and specimen stored in the National Herbarium of Addis Ababa University with a voucher number MA001. The collected plant material was air dried and grinded to a uniform size using an electric grinder.

### Extraction

Ground roots of *O. cufodontii* (300 g) were successively extracted on maceration with each 1.5 L of *n*-hexane, EtOAc and MeOH for 72 h following the standard procedure in the literature with slight modifications^16^. Each extracts was filtered and concentrated under vacuum using rotary evaporator at 40 °C to furnish 1.5, 1.2 and 3%, respectively. Likewise, the leaves (300 g) were also extracted following same procedure as above to afford 2, 1.5 and 4% of the *n*-hexane, EtOAc, and MeOH extract respectively.

### Isolation of compounds

The EtOAc extract (3 g) of the leaves of *O. cufodontii* was adsorbed and fractionated over silica gel (150 g) column chromatography with *n*-hexane:EtOAc of increasing polarities as eluent to furnish 58 fractions, each 50 mL. Fraction eluted with *n*-hexane:EtOAc (7:3) gave compound **1** (40 mg).

The *n*-hexane extract (3.0 g) of the roots of *O. cufodontii* was also fractionated over silica gel (150 g) column chromatography with increasing polarity of EtOAc in *n*-hexane as eluent to give 24 fractions, each 100 mL. Fraction 4 eluted with *n*-hexane:EtOAc (85:15) furnished compound **2** (32 mg) as yellow crystals. Fraction 10 eluted with *n*-hexane:EtOAc (4:1) is identified as compound **3** (32 mg).

The EtOAc extract (3.0 g) of the roots of *O. cufodontii* was adsorbed and subjected to silica gel (150 g) column chromatography with increasing polarities of EtOAc in *n*-hexane as eluent to give 41 fractions, each 50 mL. Fraction 7 eluted with *n*-hexane:EtOAc (9:1) afford compound **4** (30 mg). On the other hand, fraction 37 and 38 eluted with *n*-hexane:EtOAc (3:7) were merged to give compound **5** (23 mg).

### Extraction of essential oil from the leaves

The ground leaves of *O. cufodontii* (60 g) were hydro-distilled in a Clevenger’s apparatus for 4 h. The less dense yellowish oil separated using separatory funnel was dried over anhydrous Na_2_SO_4_, and kept in refrigerator until analysis. Experiments were conducted in triplicate.

### GC–MS analyses of the essential oil of the leaves of *O. cufodontii*

GC–MS analysis of the essential oil was carried out by following the method described in Hema^17^. A GC–MS instrument from Agilent Technologies (Santa Clara, CA, USA) equipped with a 6890 N network GC system, 5975 inert mass selective detector, 7683B series auto sampler injector (10 μL in size), G1701DA GC/MSD Chem Station and HP5MS column (30 m length × 0.25 mm internal diameter × 0.25 μm film thickness) coated with 5% phenyl 95% methyl poly siloxane was used for analyzing the samples. 2 μL essential oil solutions in chloroform was injected through auto sampler and analyzed with HP5MS column. Column temperature was programed as follows: 55–120 °C at 20 °C/min, 120–150 °C at 1.5 °C/min, 150–250 °C at 20 °C/min, 250 °C (10 min) and 3 min solvent delay. The mass spectra transfer line temperature was 280 °C. The carrier gas was helium (1 mL/min) with a split ratio equal to 100:1. The mass spectra were recorded in electron ionization mode at 70 eV with scanning from 50 to 500 amu (atomic mass unit) at 0.5 s with the mass source being set at 230 °C. The relative % amount of each component was calculated by comparing its average peak area to the total area Identification of the constituents was done with the aid of NIST 2005 library of mass spectra.

### Antibacterial activity

The antibacterial activity of the extracts and isolated compounds were tested using Mueller Hinton agar medium following previously reported protocol with slight modification^15^. The bacterial strains including *Escherichia coli, Klebsella pneumonia, Salmonella typhymurium* and *Staphylococcus aureus* were the American type culture collection obtained from Oromia Public Health Research, Capacity Building and Quality Assurance Laboratory Center, Adama, Ethiopia. Wells have been made in nutrient agar plate using cork borer (6 mm diameter) and inoculums containing 10^6^ CFU/mL of bacteria have been spread on the solid plates with a sterile swab moistened with the bacterial suspension and each 50 µg/mL of the extracts and isolated compounds were filled in the wells with the help of micropipette. Plates have been left for some time till the samples diffuse in the medium with the lid closed and incubated at 37 °C for 24 h. After overnight incubation, the plates were observed for the zone of inhibition (ZI) and the diameter of the inhibition zone was measured using scale. Then the samples were analyzed in triplicates and expressed as mean ± SD. Ciprofloxacin and DMSO were used as positive and negative controls, respectively.

### Molecular docking studies of the isolated compounds

AutoDock Vina with previously reported protocol^17^ was used to dock the proteins (PDB ID: 6F86) and isolated compounds (**1–5**) into the active site of proteins^18,19^. The chemical structures of compounds **1–5** were drawn using ChemOffice tool (Chem Draw 16.0) assigned with proper 2D orientation, and energy of each molecule was minimized using ChemBio3D. The energy minimized ligand molecules were then used as input for AutoDock Vina, in order to carry out the docking simulation. The crystal structure of receptor molecule *E. coli* gyraseB (PDB ID: 6F86) were downloaded from protein data bank. The protein preparation was done using the reported standard protocol^20^ by removing the co-crystallized ligand, selected water molecules and cofactors. Next, the target protein file was prepared by leaving the associated residue with protein by using Auto Preparation of target protein file Auto Dock 4.2 (MGL tools1.5.6). The graphical user interface program was used to set the grid box for docking simulations. The grid was set so that it surrounds the region of interest in the macromolecule. The docking algorithm provided with Auto Dock Vina was used to search for the best docked conformation between ligand and protein. During the docking process, a maximum of nine conformers were considered for each ligand. The conformations with the most favorable (least) free binding energy were selected for analyzing the interactions between the target receptor and ligands by Discovery studio visualizer and PyMOL. The ligands are represented in different color, H-bonds and the interacting residues are represented in ball and stick model representation.

### Antioxidant activity

#### 2,2-Diphenyl-1-picrylhydrazyl (DPPH) radical scavenging assay

The radical scavenging activity of the samples were estimated using 2,2-diphenyl-2-picrylhydrazyl (DPPH) assay^21^. Briefly, the methanol solution of the hexane extract was serially diluted with 0.004% DPPH in methanol to furnish 200, 100, 50, and 25 µg/mL of the extract. After 30 min incubation in an oven at 37 °C, absorbance at 517 nm was measured using UV–Vis spectrophotometer. Ascorbic acid and 0.004% DPPH in methanol were used as positive control and blank, respectively. The percentage inhibition of the extracts was calculated using the following formula. % Inhibition = (A_control_ − A_extract_)/A_control_ × 100 where Acontrol is the absorbance of 0.004% DPPH in methanol and Aextract is the absorbance of DPPH solution plus sample. The DPPH radical scavenging activity of the samples was also expressed as IC_50_, the concentration of the test compound to give a 50% decrease of the absorbance from that of the control solution. The radical scavenging activity of the other extracts and the isolated compounds of the leaves and the roots of *O. cufodontii* were also evaluated following the same. Ascorbic acid and quercetin were used as positive controls.

#### Ferricthiocyanate method

The lipid peroxidation inhibitory potential of the samples was evaluated using ferricthiocyanate method^22^. In brief, the hexane extract (0.1 mg) of the leaves of *O. cufodontii*, linoleic acid (100 µL), EtOH (5 mL) and phosphate buffer (5 mL, 0.05 M, pH = 7) in water were mixed and incubated at 40 °C in an oven. After 24 h, 0.1 mL was taken and added in to a vial containing 75% aqueous EtOH (7 mL), 30% of NH_4_SCN (0.15 mL) and 0.15 mL of 0.02 M FeCl_2_ in 3.5% HCl. The absorbance of the red coloured solution was measured at 500 nm using UV–Vis spectrophotometer. This was repeated every 24 h until the control gave its maximal absorbance value. Likewise, the lipid peroxidation inhibitory activity of the EtOAc extract of the leaves, the hexane and EtOAc extracts of the roots, and isolated compounds were assessed following the same procedure. Absorbance of the blank and ascorbic acid was measured in the same fashion. The percentage inhibition using ferricthiocyante method has been calculated according to the following formula.

$${\text{Percentage inhibition}} = 100 - \left( {\frac{{{\text{As}}}}{{{\text{Ab}}}} \times 100} \right)\% ,$$

where As is absorbance of the sample and Ab is absorbance of the blank^23^.

#### Ferric-reducing antioxidant power (FRAP) assay

The ferric reducing antioxidant power assay of the extracts and isolated compounds were measured according to Birasuren et al.^24^. The FRAP reagent was prepared by mixing acetate buffer (25 mL, 0.3 M), TPTZ (2,4,6-tripyridyl-s-triazine) solution (2.5 mL), and FeCl_3_∙6H_2_O solution (2.5 mL), and then heated to 37 °C before use. A properly diluted sample (0.1 mL, 100 µg/mL) was mixed with FRAP reagent (4.0 mL) to form a mixture. This was incubated at 37 °C for 10 min in the dark, and the absorbance was measured at 593 nm against a blank (distilled water). The results were expressed as aqueous solution of ferrous sulfate (FeSO_4_∙7H_2_O), and derived from a calibration curve of the standards. Likewise, the FRAP value of ascorbic acid was obtained by the same procedure.

### Statistics data analysis

The antibacterial and antioxidant assay data generated by triplicate measurements were reported using mean ± standard deviation. Analyses were performed using GraphPad Prism version 5.00 for Windows, (GraphPad Software, San Diego California USA, www.graphpad.com”). Groups were analyzed for significant differences using a linear model of variance analysis (ANOVA) test for comparisons, with level significance at p < 0.05 (Supplementary Information).

## Results and discussion

### Characterization of isolated compounds

**Figure 1:**
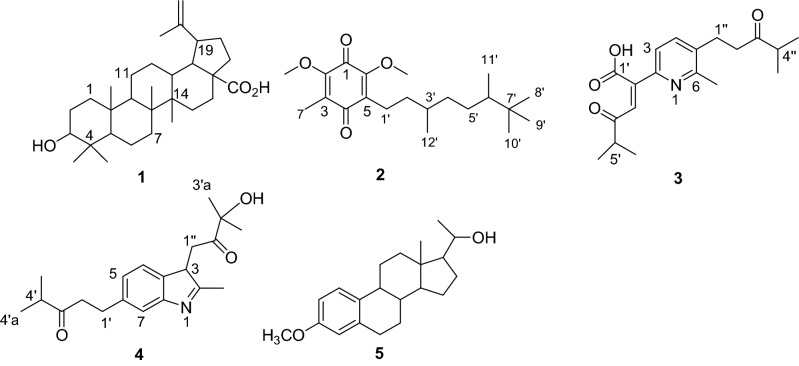
Structures of compounds 1–5 drawn by ChemDraw Pro 16.0 Suite (PerkinElmer, USA).

In the present work, five compounds from the roots and leaves of *O. cufodontii* were isolated and characterized (Fig. 1). The structure elucidations of these compounds are described herein.

Compound **1** (40 mg) was isolated as a white solid melting at 295–296 °C. TLC showed a spot at Rf 0.47 with *n*-hexane:EtOAc (7:3) as eluent which was similar to the one reported for betulinic acid^25^. The mass spectrum of **1** displayed a molecular ion peak (M−H)^+^ at m/z 455 corresponding to the formula C_30_H_48_O_3_. The ^1^H-NMR (400 MHz, CDCl_3_) spectrum of **1** showed signals attributable to an exo-methylene group at *δ* 4.64/4.51 (2H, *d*, *J* = 2.0, H-29) which together with an allylic methyl at *δ* 1.61 (3H, *s*, Me) indicated an isopropenyl function. The spectrum also showed signals for five tertiary methyl groups at *δ* 1.18, 0.74, 0.67, 0.87 and 0.67, one isopropenyl moiety at *δ* 1.61, 4.51 and 4.64, indicating a lupane-type skeleton. The signals at *δ* 1.18 (3H, *s*) and *δ* 0.74 (3H, *s*) were due to methyl protons at C-23 and C-24, respectively. In addition, the spectrum also showed a multiplet at *δ* 2.93 (1H, *m*, H-19) and 1.50 (1H, *m*, H-18). These spectral features are in close agreement to those reported for betulinic acid^26^. The ^13^C-NMR spectrum of compound **1** displayed the presence of 30 carbon signals of which six methyl, six quaternary, one carboxylic acid, eleven methylene, and six methine, confirming that compound **1** is betulinic acid^27^.

Compound **2** (32 mg) was isolated as a yellow solid from *n*-hexane extract of the root of *O. cufodontii*. The MS spectrum revealed the molecular ion peak *m/z* 345 [M + H]^+^ attributed to the molecular formula C_21_H_29_O_4_. The UV–Vis spectrum (MeOH) showed absorption maxima at 140, 240 and 290 nm suggesting a quinone skeleton^28^. The ^1^H-NMR spectral data revealed the presence of two methoxy signals *δ* 3.90 and 3.91. The absence of methine proton signals in the olefinic region justify that the carbons in this regions are all quaternary. The spectrum also displayed signals due to methylene protons at *δ* 1.33, 1.47, 1.94 and 1.98. Four methyl signals are evident at *δ* 0.92, 0.99, 1.11, 1.17, 1.59 and 1.69. The ^I3^C-NMR spectral data together with DEPT-135 revealed the presence of twenty one carbon resonances: two carbonyls (*δ* 189.7 and *δ* 184.2), two oxygenated quaternary carbons (*δ* 157.1 and 150.1), two aliphatic methine carbon (*δ* 33.0 and 41.2), three non-oxygenated quaternary carbons (*δ* 140.4, *δ* 135.6 and 45.9), two methoxy carbons (*δ* 60.7 and *δ* 62.7), four methylene carbons (*δ* 18.9, *δ* 35.9, *δ* 33.2 and *δ* 41.2), and six methyls at *δ* 18.6, 20.3, 20.5, 21.7, 26.0 and 26.1. The spectral data of **2** agreed well with the quinone whose structure is depicted in Fig. 1.

Compound **3** (38 mg) was isolated as a red crystals melted at 85–86 °C. The MS spectrum revealed the molecular ion peak at *m/z* 330.2 [M−H] ^+^ attributed to the molecular formula C_19_H_25_O_4_N. The UV–Vis spectrum (Methanol) showed absorption maxima at 340 and 540 nm, indicating the presence of conjugated chromophore in the structure. The ^1^H-NMR spectrum (400 MHz, CDCl_3_) showed signals due to four methyls at *δ* 1.12 (6H, *d*, *J* = 6.94 Hz, H-5″, 6′) and *δ* 1.08 (6H, *d*, *J* = 6.77 Hz, H-6′, 7′). Another methyl group is observed at *δ* 2.32 (3H, *s*, H-6a). The signals due to two methylene protons were apparent at *δ* 3.12 (2H, *t*, *J* = 6.84, H-1″) and *δ* 2.67 (2H, *t*, *J* = 6.84, H-2″). Signals due to three olefinic protons were evident at *δ* 7.07 (1H, *s*, H-3′), 7. 04 (1H, *d, J* = 8.42 Hz, H-3), and 7.35 (1H, *d*, *J* = 8.42 Hz, H-4). The ^13^C-NMR spectrum (Table 1) together with DEPT-135 NMR spectrum revealed the presence of seven quaternary, five methine, two methylene, and five methyl groups. The presence of three carbonyls are also evident at *δ* 213.6 (C-3″), 181.2 (C-1′) and 182.3 (C-4′). The two methylenes were observed at *δ* 24.9 (C-1″) and *δ* 38.5 (C-2″). The spectrum also displayed signals due to quaternary carbons at *δ* 147.0 (C-2), 144.7 (C-6), 134.9 (C-2′), and *δ* 128.4 (C-5). The signal due to two methine group on aromatic ring were evident at *δ* 128.5 (C-3) and *δ* 137.0 (C-4). The data generated enable us to propose the structure of compound **3** as shown in Fig. 1.

**Table 1:** ^1^H (400 MHz, CDCl_3_) and ^13^C NMR (100 MHz, CDCl_3_) data of compounds 3.

No	*δ*_H_ NMR data (*δ* in ppm, J in Hz)	*δ*_C_ NMR data (*δ* in ppm)
6a	2. 3 (3H, *s*)	19.7
2	–	147.0
3	7.04 (1H, *d*, *J* = 8.42 Hz)	128.5
4	7.35 (1H, *d, J* = 8.42 Hz)	137.0
5	–	128.5
6	–	144.7
1′	–	181.2
2′	–	134.9
3′	7.04 (1H, *s*)	140.1
4′	–	182.3
5′	2.70 (1H, *septet*)	40.7
6′	1.08 (3H, *d*, *J* = 6.77 Hz)	21.4
7′	1.08 (3H, *d*, *J* = 6.77 Hz)	21.4
1″	3.19 (2H, *t*, *J* = 6.81 Hz)	24.9
2″	2.67 (2H,t)	38.5
3″	–	213.6
4″	2.98 (1H, *septe*t)	26.9
5″	1.13 , 6H, *d*, *J* = 6.94 Hz)	18.2
6″	1.13 , 6H, *d*, *J* = 6.94 Hz)	19.7

δ in ppm and J in Hz.

The structure of compound **3** was further confirmed using 2D NMR. The COSY spectrum clearly indicates a correlation between the proton at *δ* 7.04 and *δ* 7.34. Also observed correlation in the COSY spectrum is the proton at *δ* 2.98 with 1.12 (H-5′ with H-6′, 7′) and *δ* 2.67 with 1.08 (H-4″ with H-5″, 6″). The HSQC spectrum showed the proton signals at *δ* 7.34, 7.07, 7.04, 3.19, 2.98, 2.70, 2.67, 2.30, 1.12 and 1.08 correlate with the carbon signals at *δ* 137.0, 140.1, 128.5, 24.9, 26.9, 40.7, 38.5, 19.7, 21.4 and 18.2, respectively. In the HMBC spectrum, the proton signal at *δ* 1.08 correlates with the carbon *δ* 40.7, 213.6 and 24.9. The signal at *δ* 2.7 correlates with the carbon signals at *δ* 24.9 and 213.6 (Fragment 1). The spectrum also revealed a correlation between the proton at *δ* 1.12 with the carbon at *δ* 26 and 181 (Fragment 2). While the proton at *δ* 2.98 correlates with the carbon at *δ* 181.0.
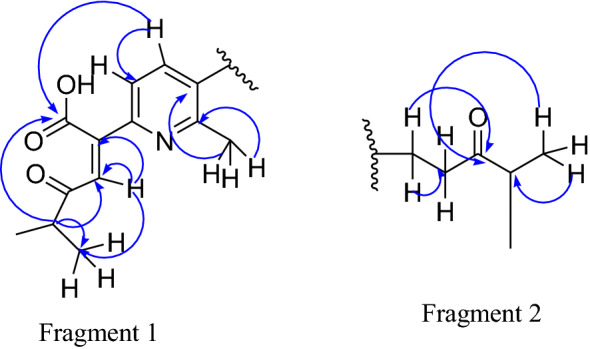


Correlations are also observed in the HMBC spectrum due to the proton at *δ* 7.04 with the carbon at *δ* 140.2 and 128. The proton at *δ* 7.34 correlates with the carbon at *δ* 147, 134, and 19. Therefore, compound **3** is a novel compound isolated for the first time from the roots of *O. cufodontii* with its structure depicted in Fig. 1.

Compound **4** (29 mg) was isolated as a brown solid from the EtOAc extract of the root of *O. cufodontii*. The mass spectrum revealed the molecular ion peak at *m/z* 330.3 [M]^+^ attributed to the molecular formula C_20_H_28_O_3_N. The UV–Vis (Methanol) showed absorption maxima at 240 and 340 nm, signifying the presence of conjugated chromophore in the structure. In the ^1^H-NMR spectrum (400 MHz, CDCl_3_) of **4,** signals due to two methyl groups at *δ* 1.21 (3H, d, H-4′a) and 1.25 (3H, *d*, 4′b) along with the signal due to methine proton at *δ* 1.21 (1H, *septet*, H-4′) suggests the presence of isopropyl group. The presence of two methyl groups on quaternary carbons are evident at *δ* 1.04, 0.87 (6H, *s*, H-3″a, 3″b). The protons at *δ* 7.21 (1H, *d*, *J* = 7.6 Hz, H-4), *δ* 7.08 (1H, *d*, *J* = 0.8 Hz, H-7) and *δ* 7.04 (1H, *d*, *J* = 7.6 Hz, H-5) indicate the presence of a 1,3-disubsituted aromatic ring. Three methylene protons are evident at *δ* 1.40 (2H, *m*, H-1′), *δ* 1.98 (2H, *m,* H-2′) and *δ* 3.00 (2H, *m*, H-1″). The spectrum also displayed signal due to methine proton and methyl protons at *δ* 2.90 (1 H, *m,* H-3) and 2.40 (3 H, *s,* H-2a), respectively.

The proton decoupled ^13^C-NMR spectrum with the aid of DEPT-135 revealed the presence of seven quaternary, five methine, three methylene, and five methyl groups. The spectrum (Table 2) revealed the presence of two carbonyl carbons at *δ*_C_ 210.5 (C-3′) and 203.5 (C-2″). The three methylene groups are apparent at *δ* 40.3 (C-2′), 22.0 (C-1′) and 29.7 (C-1″). The sp2 quaternary carbons at *δ* 140.5, 138.3, 137.9 and 121.1 were attributed to C-2, C-8a, C-6 and C-8, respectively. The other oxygenated sp3 quaternary carbon appeared at *δ* 81.9 (C-3″). Two methine carbons are also evident at *δ* 27.3 (C-4′) and 55.7 (C-3). The data generated led us to the identification of compound **4** as shown in Fig. 1.

**Table 2:** ^1^H (CDCl_3_, 400 MHz) and ^13^C NMR (CDCl_3_, 100 MHz) data of compounds 4.

No	^1^H-NMR data of compound 4	^13^C-NMR data of compound 4
2	–	140.5
2a	2.40 (3 H, *s,* H-2a)	17.8
3	2.90 (1 H, *m,* H-3)	55.7
4	7.21 (1H, *d*, *J* = 7.6 Hz, H-4),	141.2
5	7.04 (1H, *d* , *J* = 7.6 Hz, H-5)	130.8
6	–	137.9
7	7.08 (1H, *d*, *J* = 0.8 Hz, H-7)	127.6
8	–	138.3
8a	–	129.1
1′	1.40 (2 H, *m*, H-1′)	22.0
2′	1.98 (2 H, *m*, H-2′)	40.3
3′	–	210.5
4′	1.25 (1H, *septet*, H-4′)	27.3
4′a	1.21 (3H, *d*, *J* = 7.2 Hz, H-4′a)	18.9
4′b	1.25 (3H, *d*, *J* = 7.2 Hz, H-4′b)	18.2
1″	3.00 (2 H, *d*, *J* = 2.4 Hz,H-1″)	29.7
2″	–	203.5
3″	–	81.9
3″a	1.04 (3H, *s,* H-3″a)	21.4
3″b	0.87 (3H, *s*, H-3″b)	21.5

Compound **5** (23 mg) was isolated as a white powder from the ethyl acetate extract of the root of *O. cufodontii*. The UV–Vis spectrum showed absorption band at 340 nm indicating the presence of conjugated chromphore in the compound. The ^1^H-NMR spectrum (CDCl_3_, 400 MHz) revealed the presence of the three aromatic protons at *δ* 7.90 (1H), *δ* 7.75 (1H), and 7.63 (1H). The spectrum showed the presence of a methoxy group at *δ* 3.85 (3H, *s*). The signal observed at *δ* 3.41 (1H, m) is due to the proton on carbon bearing oxygen*.* The ^13^C-NMR spectrum (CDCl_3_, 100 MHz) show 21 carbon signals which are in agreement with the number of carbon atoms in this compound. The signals due to aromatic protons were evident at 148.4, 145.8, 139.9, 135.9, 124.5, and 116.0. The signals due to methoxy group and an oxygenated aliphatic carbon were observed at *δ* 61.9 and 77.2, respectively. The spectrum also showed signals in the aliphatic regions at* δ* 14.1, 22.2, 23.4, 23.6, 27.2, 29.7, 30.71, 30.72, 30.73, 38.4, 38.9, 41.8, and 44.0. The data generated led us to the identification of compound 5 as a known compound whose structure is depicted in Fig. 1.

### Essential oil analysis

Hydro-distillation of the air dried ground leaves of *O. cufodontii* gave a yellow liquid essential oil with a yield of 2.66% w/w. The yield obtained herein is superior compared with the essential oil obtained from *O. gratissimum*^21^. The presence of such huge quantities of essential oil from the leaves of *O. cufodontii* may justify the uses of this species as spices by the local people. The GC–MS analysis of the essential oil has led to the identification of *β*-caryophyllene as a principal component with 31.3%. Such significant amount of *β*-caryophyllene was also reported from the essential oil of *O. campechianum*^29^. The other constituents identified in the essential oil of *O. cufodontii* are *Z*,*Z*,*Z*-1,4,6,9-nonadecatetraene (30.64%), methyl-10,12-pentacosadiynoate (10.97%), 8,11-Octadecadiynoic acid, methyl ester (9.21%), 6,9,12-Octadecatrienoic acid, and phenylmethyl ester, (*Z*,*Z*,*Z*)-(5.57%). The presence of huge quantities of *β*-caryophyllene indicates the essential oils of *O. cufodontii* to be used as medicine and spices to enhance the taste of food.

### Antibacterial activity

The findings of the in vitro antibacterial activity of the extracts and isolated compounds from the leaves and roots of *O. cufodontii* were presented in Table 3.

**Table 3:** Inhibition zone (in mm) displayed by constituents of the leaves and roots of *O. cufodontii* at dose of 50 µg/mL.

Samples	*S. aureus*	*E. coli*	*K. phnemonea*	*S. typhmurium*
HLE	12.01 (0.20)	8.00 (0.10)	6.00 (0.30)	7.02 (0.10)
EALE	8.00 (0.10)	6.02 (0.09)	8.01 (0.09)	6.00 (0.10)
MLE	8.02 (0.10)	6.01 (0.19)	6.02 (0.10)	6.00 (0.30)
HRE	17.09 (0.30)	8.01 (0.10)	7.02 (0.10)	9.03 (0.09)
EARE	13.00 (0.20)	6.01 (0.30)	8.03 (0.08)	10.01 (0.10)
MRE	15.04 (0.10)	6.02 (0.30)	6.03 (0.10)	6.01 (0.20)
EO	19.04 (0.10)	6.01 (0.20)	8.03 (0.50)	8.00 (0.10)
1	12.02 (0.20)	9.02 (0.30)	8.03 (0.10)	10.03 (0.30)
3	14.01 (0.30)	10.02 (0.20)	7.01 (0.10)	9.00 (0.10)
4	15.02 (0.10)	11.01 (0.30)	9.02 (0.40)	13.03 (0.10)
DMSO	0	0	0	0
Ciprofloxacin	19.30 (0.10)	19.01 (0.67)	22.00 (0.10)	21.50 (0.30)

Samples were analyzed in triplicates and expressed as m ± SD.

*HLE* hexane leaves extract, *EALE* EtOAc leaves extract, *MLE* MeOH leaves extract, *HRE* hexane root extract, *ERE* EtOAc root extract, *MRE* MeOH root extract, *EO* Essential oil.

As clearly depicted in Table 3, the extracts and isolated compounds from the roots and leaves of *O. cufodontii* show differences in the inhibition of growth of bacterial strains. The absolute values of the diameter zones of inhibition (DZI) varied from 6.00 to 19.04 ± 0.10 mm. The hexane extract of the leaves of *O. cufodontii* displayed DZI of 12 0.01 mm while the EtOAc and MeOH extract showed DZI of each 8.00 mm indicating that the active constituents are likely residing in the non-polar fraction of the leaves of *O. cufodontii*. Likewise the hexane, EtOAc and MeOH extract of the roots exhibited DZI of 17.09, 13.00 and 15.04 mm against *S. aureus*, respectively. The results displayed by the roots extracts are superior compared with that of the leaves of *O. cufodontii.* Compounds 1, 3 and 4 were also evaluated for their antibacterial activity and better inhibition was observed against *S. aureus* with DZI of 12.02, 14.01, and 15.02 mm, respectively. The latter exhibited good activity with both Gram positive and gram negative bacteria. Therefore, the antibacterial activity displayed by the extracts of roots of *O. cufodontii* reveals the presence of compound **3** and **4**.

Moreover, the essential oil extracted from the leaves displayed significant antibacterial activity against *S. aureus* with DZI of 19.04 ± 0.1 mm. The activity demonstrated by the hexane extract of the leaves might be due to the presence of essential oils in the non-polar fraction of *O. cufodontii.* Under the same conditions, the EO exhibited similar antibacterial activity with chloramphenicol used as positive control for same strain. The antibacterial activity of the essential oil extracted from the leaves of *O. cufodontii* was also compared with the literature reported for essential oil from *O. gratissimum* and the results are almost similar^30^. The zone of inhibition of the extracts and isolated compounds of the roots extract of *O. cufodontii* show that the plant has good potential as a remedy for diseases caused by gram negative bacterial pathogens. Furthermore, results obtained from the present work corroborate the traditional use of the roots of *O. cufodontii* against bacteria.

### Antioxidant activity

DPPH assay is a simple method to assess antioxidants activities by measuring absorbance at 517 nm due to the formation of DPPH radical^29,31^. The extracts and isolated compounds from the leaves and roots of *O. cufodontii* change the purple colored DPPH radical to the yellow-colored diphenylpicrylhydrazicine. The extracts also reduce the absorbance of the DPPH radical at 517 nm indicating their potential as radical scavengers. The activity increases in a dose dependent manner. The hexane extract of the roots of *O. cufodontii* reduced the DPPH radical by 90.50% at 200 µg/mL (Table 4). This is superior to the activity displayed by the other extracts. This is evident from the low IC_50_ (4.4 µg/mL) value observed for the hexane extract while the IC_50_ value of the hexane extract of the leaves, the EtOAc extract of the leaves and the roots were 6.0, 48 and 122 µg/mL, respectively. This is in agreement with the high radical scavenging activity reported in the literature for the hexane extracts of various non-pungent peppers compared with the EtOAc and methanol extracts^32^. Ascorbic acid and quercetin used as positive control scavenged the DPPH radical by 93.02 and 89.01% at 100 µg/mL, respectively while their corresponding IC_50_ values were 3.45 and 4.41 µg/mL. The result was comparable to the activity displayed by the hexane extract of the roots of *O. cufodontii.* Literature reports have shown that *O. gratissimum, O. americanum, O. minimum* and *O. citriodorum* had radical scavenging activity of 81.1, 77.4, 70.1 and 60.6%, respectively^33^. This indicates that the activity displayed by the hexane extract of the roots of *O. cufodontii* is superior than the radical scavenging properties of its sister species.

**Table 4:** DPPH radical scavenging and lipid peroxidation inhibitory activities of extracts and constituents of the roots and leaves of *O. cufodontii*.

Samples	%DPPH inhibition at					Anti-lipid peroxidation % inhibition
	200 (µg/mL)	100 (µg/mL)	50 (µg/mL)		25 (µg/mL)	
HLE	65.02 (0.04)	57.90 (0.20)	52.81 (0.03)		51.72 (0.09)	70.01 (0.08)
EALE	68.32 (0.01)	53.21 (0.09)	50.73 (0.07)		48.50 (0.11)	52.02 (0.12)
HRE	90.50 (0.03)	85.01 (0.05)	61.22 (0.08)		50.90 (0.08)	83.02 (0.01)
EARE	52.44 (0.01)	49.23 (0.01)	48.81 (0.02)		45.92 (0.20)	57.01 (0.70)
1	32.30 (0.03)	28.52 (0.06)	24.00 (0.10)		19.21 (0.09)	22.01 (0.19)
3	39.22 (0.31)	35.81 (0.90)	28.22 (0.05)		16.80 (0.40)	76.02 (0.80)
4	37.90 (0.27)	32.70 (0.50)	26.90 (0.10)		22.82 (0.20)	69.01 (0.10)
Ascorbic acid	93.02 (0.02)	80.02 (0.04)	60.02 (0.05)		50.09 (0.02)	87.08 (0.02)
Quercetin	89.01 (0.06)	73.00 (0.10)	64.03 (0.21)		64.01 (0.08)	85.02 (0.07)

*HLE* hexane leaves extract, *EALE* EtOAc leaves extract, *HRE* hexane root extract, *EARE* EtOAc root extract; Samples were reported as mean ± SEM; Ascorbic acid and quercetin used as positive control were measured at 100, 50, 25 and 12 µg/mL.

Compounds **1**, **3** and **4** inhibited DPPH radical by 32.30, 39.22, and 37.90%, respectively. As revealed from Table 4, the DPPH radical scavenging activities of the isolated compounds are inferior compared with the activity shown by extracts. This is likely ascribed to the presence of phenols and flavonoids in the extracts of the roots and leaves of *O. cufodontii.* The presence of flavonoids was further confirmed by the formation of yellow orange color on treating the extracts of the roots and leaves of *O. cufodontii* with NaOH followed by HCl^34^. Moreover, the extracts furnished a bluish green color on treatment with FeCl_3_ supporting the presence of phenols in the extracts^35^.

The lipid peroxidation inhibitory potential of the extracts and isolated compounds were assessed using ferricthiocyante methods with the results presented in Table 4. As observed from Table 4, the hexane extracts of the leaves and the roots inhibited peroxide formation by 70.01 and 83.02%, respectively. The activity displayed by the latter extract is better than the activity displayed by the isolated compounds. The activity displayed by the hexane extract of the roots of *O. cufodontii* is comparable with the standard drug demonstrating the lipid peroxidation inhibitory potential of the hexane extract. This indicates the potential use of the extracts of the leaves and roots of *O. cufodontii* as remedy against diseases caused by radicals.

### Ferric-reducing antioxidant power (FRAP) assay

FRAP assay is used to evaluate the antioxidant potential of extracts based on the reduction of the Fe^3+^-TPTZ (2,4,6-tripyridyl-s-triazine) complex to the ferrous form at a pH around 3.6^24^. The ferric-TPTZ complex can be monitored at 593 nm^36^. In view of this, the ferric-reducing antioxidant power of the extracts and isolated compounds was assessed and the results are depicted in Table 5.

**Table 5:** Ferric-reducing potential of extracts and isolated compounds of *O. cufodontii*.

Samples	mmol Fe(II)/mg of samples
HLE	0.090 (0.004)
EALE	0.087 (0.003)
HRE	1.120 (0.007)
EARE	0.900 (0.006)
1	0.050 (0.009)
3	0.070 (0.009)
4	0.060 (0.001)
Ascorbic acid	1.250 (0.002)

Samples were measured in triplicates and expressed as M ± SD; Ascorbic acid was used as positive control.

The higher absorbance of the extract indicates a higher ferric reducing power. As clearly seen from Table 5, the FRAP values for HLE, EALE, HRE and EARE were 0.090, 0.087, 1.120 and 0.900 mmol Fe(II)/mg extract. This shows that the hexane extract of the roots of *O. cufodontii* exhibited the maximum reducing power. Because the root extract of *O. cufodontii* possesses antioxidant activity, it can be vital to human health.

### Molecular docking studies

Compounds with antimicrobial activities disable bacteria by targeting key components of bacterial metabolism including cell-walls, DNA-directed RNApolymerase, protein synthesis, enzymes and DNAgyrase. The latter controls the topology of DNAduring transcription and replication by introducing transient breaks to both DNA strands^37–39^. Since DNAgyrase is pivotal for bacterial survival, it is essential to exploits bacterial DNAgyrase as a key target of antibacterial agent^39^. In view of this, the molecular docking study was carried out to examine the binding interactions of isolated compounds with the pocket of DNAgyrase and compared with ciprofloxacin. All the synthesized compounds exhibited well established bonds with one or more amino acids in the active pocket of the enzyme. The compounds also displayed minimum binding energy ranging from − 6.1 to − 6.9 kcal/mol (Table 6) with compound **3** shown to have comparable binding score and amino acid interactions compared to ciprofloxacin. The binding affinity of the synthetic compounds along with their bonding interactions of ligands (**1–5**) were summarized in Table 6.

**Table 6:** Molecular docking value of compounds isolated from root extracts of *O. cufodontii*.

Compounds	Affinity (kcal/mol)	H-bond	Residual interactions	
			Hydrophobic/Pi-Cation	Van dar Waals
**1**	− 6.3	Arg-76	Ile-78	Asp-73, Glu-50, Ile-94, Asp-49, Ieu-98, Val-120
**2**	− 6.1	–	Ile-78, Ile-94, Glu-50	Asp-73, Arg-76, Asn-46, Gly-77, Thr-165, Asp-49,
**3**	− 6.9	Asn-46, Arg-76	Ile-78, Glu-50	Asp-43, Ala-47, Gly-77, Pro-79, Val-43, Arg-136, Val-167, Thr-165
**4**	− 6.6	Asn-46	Glu-50, Ile-78	Asp-73, Asp-49, Ala-47, Gly-77, Pro-79, Ile-94, Arg-76, Val-43, Thr-165, Val-167
**5**	− 6.6	Asp-73	Arg-76	Asn-46, Ala-47, Glu-50, gly-77, Ile-78, Pro-79, Thr-165
**Ciprofloxacin**	− 6.9	Asp-73, Asn-46, Arg-76	Ile-78, Ile-94, Glu-50, Gly-77	Ala-47, Pro-79, Thr-165

**Figure 2:**
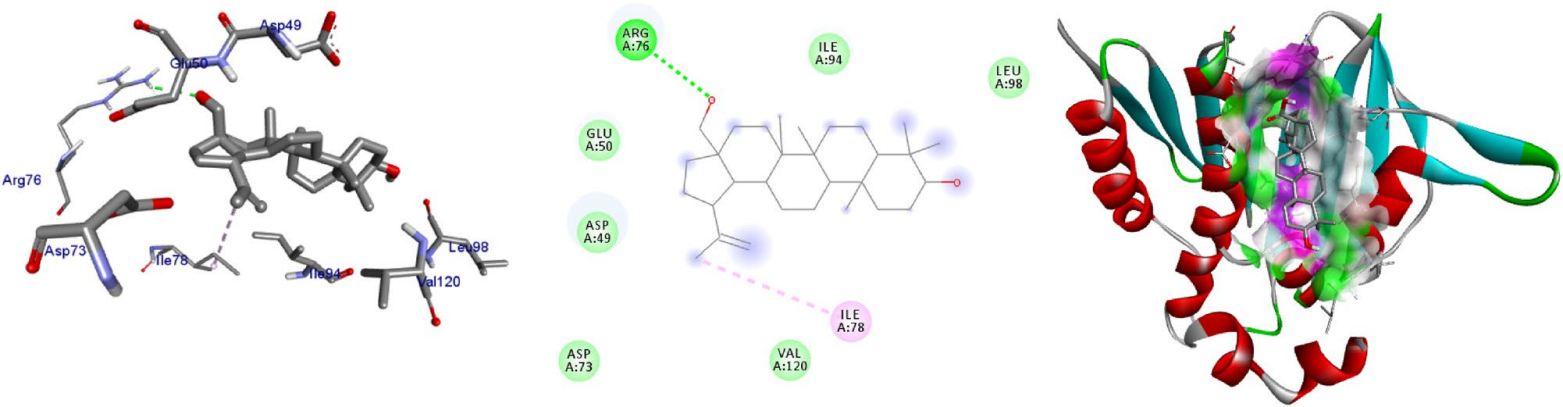
The 2D and 3D intermolecular contact between compound **1** and DNA gyrase B (PDB ID: 6F86). Chemical structures were drawn by ChemDraw Pro 16.0 Suite (PerkinElmer, USA) and analyzed by the Discovery studio visualizer (BIOVIA Discovery studio 2020 Client).

**Figure 3:**
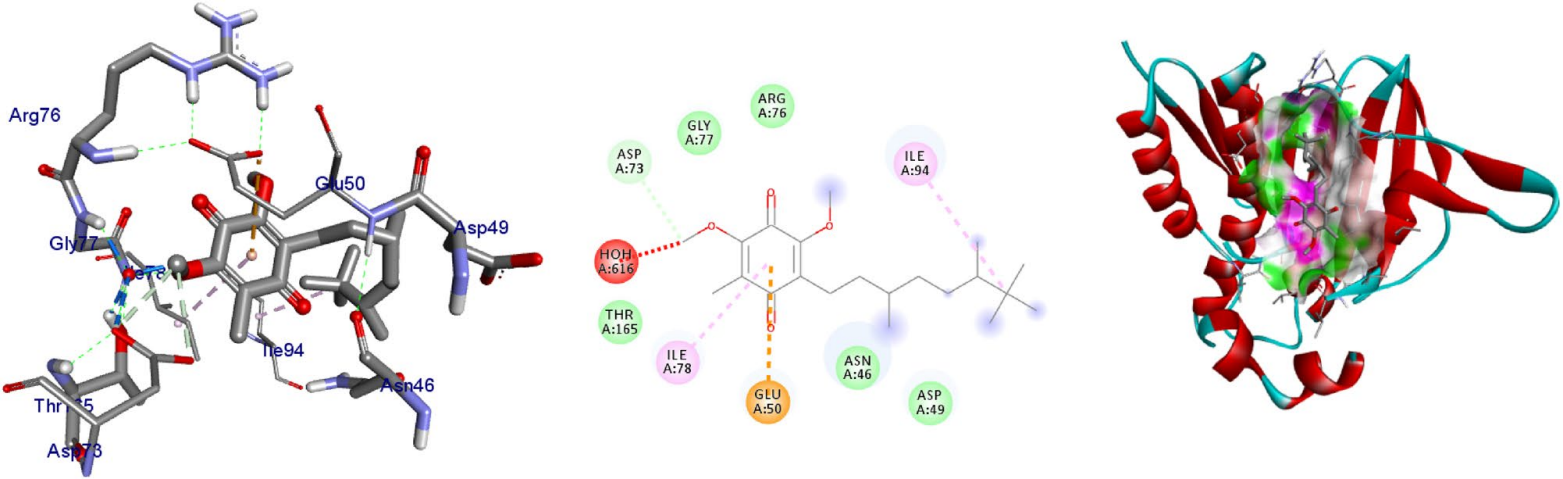
The 2D and 3D intermolecular contact between compound **2** and DNA gyrase B (PDB ID: 6F86). Chemical structures were drawn by ChemDraw Pro 16.0 Suite (PerkinElmer, USA) and analyzed by the Discovery studio visualizer (BIOVIA Discovery studio 2020 Client).

**Figure 4:**
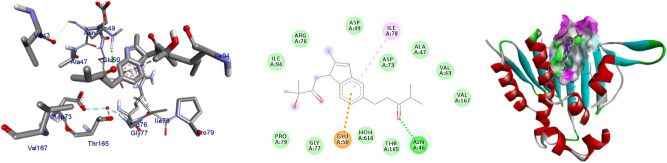
The 2D and 3D intermolecular contact between compound **4** and DNA gyrase B (PDB ID: 6F86). Chemical structures were drawn by ChemDraw Pro 16.0 Suite (PerkinElmer, USA) and analyzed by the Discovery studio visualizer (BIOVIA Discovery studio 2020 Client).

**Figure 5:**
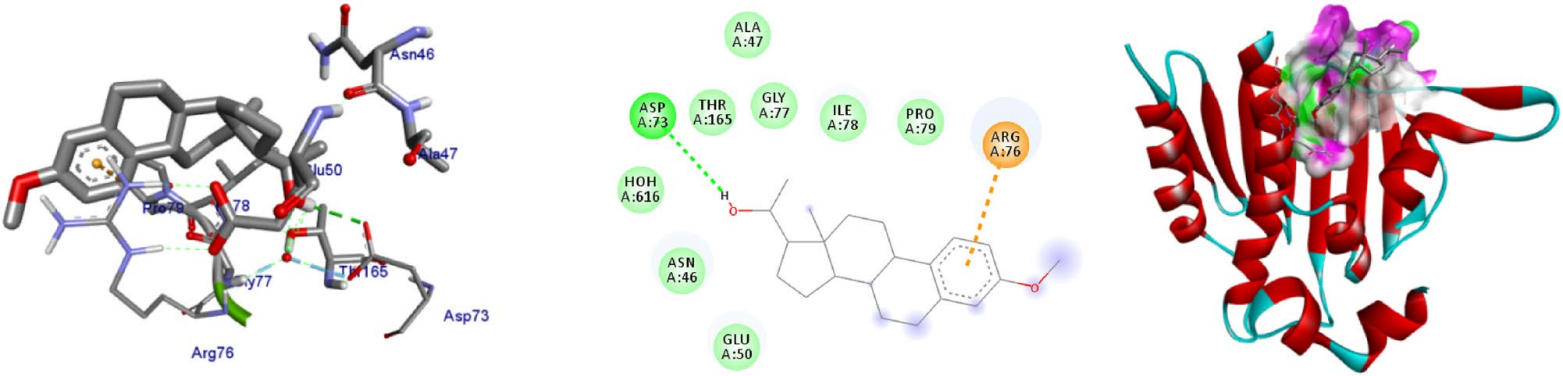
The 2D and 3D intermolecular contact between compound **5** and DNA gyrase B (PDB ID: 6F86). Chemical structures were drawn by ChemDraw Pro 16.0 Suite (PerkinElmer, USA) and analyzed by the Discovery studio visualizer (BIOVIA Discovery studio 2020 Client).

**Figure 6:**
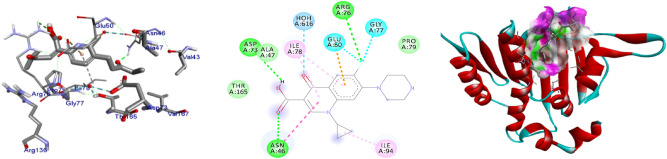
The 2D and 3D intermolecular contact between ciprofloxacin and DNA gyrase B (PDB ID: 6F86). Chemical structures were drawn by ChemDraw Pro 16.0 Suite (PerkinElmer, USA) and analyzed by the Discovery studio visualizer (BIOVIA Discovery studio 2020 Client).

The extracted natural product molecules **1–5** showed better docking efficiency with DNA gyrase B within the binding pocket. In comparison to ciprofloxacin, all the isolated compounds have shown similar residual amino acids binding interactions with Ala-47, Glu-50, Gly-77, Ile-78, Pro-79, Ile-94, Thr-165 (Hydrophobic) and Asp-73, Arg-76, Asn-46 (Hydrogen bonds). The, isolated compound **1** forms additional residual interaction with Val-120 in the active site of the target protein with the docking score − 6.3 kcal/mol. Compound **2** has the docking score − 6.1 kcal/mol with no hydrogen bond or additional residual interactions within the binding pocket. The compounds **3** and **4** each formed additional residual Van dar Waals interaction with residual amino acids Val-43 and Val-167. Compound **3** with docking score − 6.9 kcal/mol has two hydrogen bonds (Asn-46, Arg-76) and similar residual amino acid interaction within the binding pocket like standard inhibitor Ciprofloxacin. The compounds **4** and **5** both have docking score of − 6.6 kcal/mol and forms one hydrogen bond with Asn-46 and Asp-73, respectively. The Molecular docking study of these compounds displayed moderate (− 6.1) to better (− 6.9) docking score within binding pocket toward *E. coli* DNA gyrase B (6F86). Overall, in silico molecular docking analysis of the isolated compounds matches with in vitro analysis against *S. aureus.* Among compounds analyzed, compound **3** shown comparable residual interactions and docking score of ciprofloxacin. Hence, compound **3** have a better antibacterial agent than the other isolated compounds in this investigation. The binding affinity, H-bonds, and residual amino acid interactions of the five compounds are summarized in Table 5 and their binding interactions are shown in Figs. 2, 3, 4, 5 and 6. Ribbon model shows the binding pocket structure of DNA gyrase B with compounds. Hydrogen bonds between compounds and amino acids are shown as green dashed lines while hydrophobic interaction are shown as pink lines.

## Conclusion

In conclusion, two novel compounds **3** and **4** have been isolated and characterized from the roots extracts of *O. cufodontii*. The *n*-hexane extracts of the roots, the essential oil of the leaves of *O. cufodontii,* and compound **4** have shown comparable results to ciprofloxacin. The radical scavenging activity and anti-lipid peroxidation potential displayed by the *n*-hexane extracts of the roots of *O. cufodontii* also suggests the potential of the plant as an antioxidant. The in silico molecular docking studies revealed that all the isolated compounds **1–5** have close binding energy to the standard drug and may be considered as a good inhibitor of DNA gyrase. Hence, the activity displayed herein indicates that the plant has a great potential as a remedy for diseases caused by bacteria and radicals. Furthermore, the results obtained in the present study may help substantiate the traditional use of the roots of *O. cufodontii* with modern scientific based medical treatment.

## Supplementary Information

Supplementary Information.
