# Supplementary material for: Molecular docking analysis and evaluation of the antibacterial and antioxidant activities of the constituents of Ocimum cufodontii
Source: Sci Rep. 2021 May 12;11:10101. doi: 10.1038/s41598-021-89557-x (PMC8115310; doi:10.1038/s41598-021-89557-x)
Supplement: Supplementary file 1 — Supplementary Information. [file 41598_2021_89557_MOESM1_ESM.docx]

**Supplementary Information**

1. **Statistical analysis for Antibacterial assay.**

| Parameter |  |  |  |  |
| --- | --- | --- | --- | --- |
| Table Analyzed | **Antibacterial Assay** | | | |
|  |  |  |  |  |
| Two-way ANOVA |  |  |  |  |
|  |  |  |  |  |
| Source of Variation | % of total variation | P value |  |  |
| Column Factor | 62.08 | < 0.0001 |  |  |
| Row Factor | 22.46 | < 0.0001 |  |  |
|  |  |  |  |  |
| Source of Variation | P value summary | Significant? |  |  |
| Column Factor | *** | Yes |  |  |
| Row Factor | *** | Yes |  |  |
|  |  |  |  |  |
| Source of Variation | Df | Sum-of-squares | Mean square | F |
| Column Factor | 10 | 563.9 | 56.39 | 12.05 |
| Row Factor | 3 | 204.0 | 68.01 | 14.53 |
| Residual | 30 | 140.4 | 4.680 |  |
|  |  |  |  |  |
| Number of missing values | 0 |  |  |  |
|  |  |  |  |  |
| Bonferroni posttests |  |  |  |  |
|  |  |  |  |  |
| Ciprofloxacin vs aureus |  |  |  |  |
| Row Factor | Ciprofloxacin | aureus | Difference | 95% CI of diff. |
| S. aureus | 19.30 | 12.00 | -7.300 | -18.20 to 3.600 |
| E. coli | 19.00 | 8.000 | -11.00 | -21.90 to -0.1004 |
| K. phnemonea | 22.00 | 6.000 | -16.00 | -26.90 to -5.100 |
| S. typhmurium | 21.50 | 7.000 | -14.50 | -25.40 to -3.600 |
|  |  |  |  |  |
| Row Factor | Difference | t | P value | Summary |
| S. aureus | -7.300 | 2.386 | P > 0.05 | ns |
| E. coli | -11.00 | 3.595 | P<0.01 | ** |
| K. phnemonea | -16.00 | 5.230 | P<0.001 | *** |
| S. typhmurium | -14.50 | 4.739 | P<0.001 | *** |
|  |  |  |  |  |
| Ciprofloxacin vs EALE |  |  |  |  |
| Row Factor | Ciprofloxacin | EALE | Difference | 95% CI of diff. |
| S. aureus | 19.30 | 8.000 | -11.30 | -22.20 to -0.4004 |
| E. coli | 19.00 | 6.000 | -13.00 | -23.90 to -2.100 |
| K. phnemonea | 22.00 | 8.000 | -14.00 | -24.90 to -3.100 |
| S. typhmurium | 21.50 | 6.000 | -15.50 | -26.40 to -4.600 |
|  |  |  |  |  |
| Row Factor | Difference | t | P value | Summary |
| S. aureus | -11.30 | 3.694 | P<0.01 | ** |
| E. coli | -13.00 | 4.249 | P<0.001 | *** |
| K. phnemonea | -14.00 | 4.576 | P<0.001 | *** |
| S. typhmurium | -15.50 | 5.066 | P<0.001 | *** |
|  |  |  |  |  |
| Ciprofloxacin vs MLE |  |  |  |  |
| Row Factor | Ciprofloxacin | MLE | Difference | 95% CI of diff. |
| S. aureus | 19.30 | 8.000 | -11.30 | -22.20 to -0.4004 |
| E. coli | 19.00 | 6.000 | -13.00 | -23.90 to -2.100 |
| K. phnemonea | 22.00 | 6.000 | -16.00 | -26.90 to -5.100 |
| S. typhmurium | 21.50 | 6.000 | -15.50 | -26.40 to -4.600 |
|  |  |  |  |  |
| Row Factor | Difference | t | P value | Summary |
| S. aureus | -11.30 | 3.694 | P<0.01 | ** |
| E. coli | -13.00 | 4.249 | P<0.001 | *** |
| K. phnemonea | -16.00 | 5.230 | P<0.001 | *** |
| S. typhmurium | -15.50 | 5.066 | P<0.001 | *** |
|  |  |  |  |  |
| Ciprofloxacin vs HRE |  |  |  |  |
| Row Factor | Ciprofloxacin | HRE | Difference | 95% CI of diff. |
| S. aureus | 19.30 | 17.00 | -2.300 | -13.20 to 8.600 |
| E. coli | 19.00 | 8.000 | -11.00 | -21.90 to -0.1004 |
| K. phnemonea | 22.00 | 7.000 | -15.00 | -25.90 to -4.100 |
| S. typhmurium | 21.50 | 9.000 | -12.50 | -23.40 to -1.600 |
|  |  |  |  |  |
| Row Factor | Difference | t | P value | Summary |
| S. aureus | -2.300 | 0.7518 | P > 0.05 | ns |
| E. coli | -11.00 | 3.595 | P<0.01 | ** |
| K. phnemonea | -15.00 | 4.903 | P<0.001 | *** |
| S. typhmurium | -12.50 | 4.086 | P<0.01 | ** |
|  |  |  |  |  |
| Ciprofloxacin vs EARE |  |  |  |  |
| Row Factor | Ciprofloxacin | EARE | Difference | 95% CI of diff. |
| S. aureus | 19.30 | 13.00 | -6.300 | -17.20 to 4.600 |
| E. coli | 19.00 | 6.000 | -13.00 | -23.90 to -2.100 |
| K. phnemonea | 22.00 | 8.000 | -14.00 | -24.90 to -3.100 |
| S. typhmurium | 21.50 | 10.00 | -11.50 | -22.40 to -0.6004 |
|  |  |  |  |  |
| Row Factor | Difference | t | P value | Summary |
| S. aureus | -6.300 | 2.059 | P > 0.05 | ns |
| E. coli | -13.00 | 4.249 | P<0.001 | *** |
| K. phnemonea | -14.00 | 4.576 | P<0.001 | *** |
| S. typhmurium | -11.50 | 3.759 | P<0.01 | ** |
|  |  |  |  |  |
| Ciprofloxacin vs MRE |  |  |  |  |
| Row Factor | Ciprofloxacin | MRE | Difference | 95% CI of diff. |
| S. aureus | 19.30 | 15.00 | -4.300 | -15.20 to 6.600 |
| E. coli | 19.00 | 6.000 | -13.00 | -23.90 to -2.100 |
| K. phnemonea | 22.00 | 6.000 | -16.00 | -26.90 to -5.100 |
| S. typhmurium | 21.50 | 6.000 | -15.50 | -26.40 to -4.600 |
|  |  |  |  |  |
| Row Factor | Difference | t | P value | Summary |
| S. aureus | -4.300 | 1.405 | P > 0.05 | ns |
| E. coli | -13.00 | 4.249 | P<0.001 | *** |
| K. phnemonea | -16.00 | 5.230 | P<0.001 | *** |
| S. typhmurium | -15.50 | 5.066 | P<0.001 | *** |
|  |  |  |  |  |
| Ciprofloxacin vs EO |  |  |  |  |
| Row Factor | Ciprofloxacin | EO | Difference | 95% CI of diff. |
| S. aureus | 19.30 | 19.00 | -0.3000 | -11.20 to 10.60 |
| E. coli | 19.00 | 6.000 | -13.00 | -23.90 to -2.100 |
| K. phnemonea | 22.00 | 8.000 | -14.00 | -24.90 to -3.100 |
| S. typhmurium | 21.50 | 8.000 | -13.50 | -24.40 to -2.600 |
|  |  |  |  |  |
| Row Factor | Difference | t | P value | Summary |
| S. aureus | -0.3000 | 0.09806 | P > 0.05 | ns |
| E. coli | -13.00 | 4.249 | P<0.001 | *** |
| K. phnemonea | -14.00 | 4.576 | P<0.001 | *** |
| S. typhmurium | -13.50 | 4.413 | P<0.001 | *** |
|  |  |  |  |  |
| Ciprofloxacin vs 1 |  |  |  |  |
| Row Factor | Ciprofloxacin | 1 | Difference | 95% CI of diff. |
| S. aureus | 19.30 | 12.00 | -7.300 | -18.20 to 3.600 |
| E. coli | 19.00 | 9.000 | -10.00 | -20.90 to 0.8996 |
| K. phnemonea | 22.00 | 8.000 | -14.00 | -24.90 to -3.100 |
| S. typhmurium | 21.50 | 10.00 | -11.50 | -22.40 to -0.6004 |
|  |  |  |  |  |
| Row Factor | Difference | t | P value | Summary |
| S. aureus | -7.300 | 2.386 | P > 0.05 | ns |
| E. coli | -10.00 | 3.269 | P < 0.05 | * |
| K. phnemonea | -14.00 | 4.576 | P<0.001 | *** |
| S. typhmurium | -11.50 | 3.759 | P<0.01 | ** |
|  |  |  |  |  |
| Ciprofloxacin vs 3 |  |  |  |  |
| Row Factor | Ciprofloxacin | 3 | Difference | 95% CI of diff. |
| S. aureus | 19.30 | 14.00 | -5.300 | -16.20 to 5.600 |
| E. coli | 19.00 | 10.00 | -9.000 | -19.90 to 1.900 |
| K. phnemonea | 22.00 | 7.000 | -15.00 | -25.90 to -4.100 |
| S. typhmurium | 21.50 | 9.000 | -12.50 | -23.40 to -1.600 |
|  |  |  |  |  |
| Row Factor | Difference | t | P value | Summary |
| S. aureus | -5.300 | 1.732 | P > 0.05 | ns |
| E. coli | -9.000 | 2.942 | P < 0.05 | * |
| K. phnemonea | -15.00 | 4.903 | P<0.001 | *** |
| S. typhmurium | -12.50 | 4.086 | P<0.01 | ** |
|  |  |  |  |  |
| Ciprofloxacin vs 4 |  |  |  |  |
| Row Factor | Ciprofloxacin | 4 | Difference | 95% CI of diff. |
| S. aureus | 19.30 | 15.00 | -4.300 | -15.20 to 6.600 |
| E. coli | 19.00 | 11.00 | -8.000 | -18.90 to 2.900 |
| K. phnemonea | 22.00 | 9.000 | -13.00 | -23.90 to -2.100 |
| S. typhmurium | 21.50 | 13.00 | -8.500 | -19.40 to 2.400 |
|  |  |  |  |  |
| Row Factor | Difference | t | P value | Summary |
| S. aureus | -4.300 | 1.405 | P > 0.05 | ns |
| E. coli | -8.000 | 2.615 | P > 0.05 | ns |
| K. phnemonea | -13.00 | 4.249 | P<0.001 | *** |
| S. typhmurium | -8.500 | 2.778 | P < 0.05 | * |

1. **Statistical analysis for DPPH inhibition**

| Parameter |  |  |  |  |
| --- | --- | --- | --- | --- |
| Table Analyzed | **DPPH inhibition** | | | |
|  |  |  |  |  |
| Two-way ANOVA |  |  |  |  |
|  |  |  |  |  |
| Source of Variation | % of total variation | P value |  |  |
| Column Factor | 86.08 | < 0.0001 |  |  |
| Row Factor | 9.67 | < 0.0001 |  |  |
|  |  |  |  |  |
| Source of Variation | P value summary | Significant? |  |  |
| Column Factor | *** | Yes |  |  |
| Row Factor | *** | Yes |  |  |
|  |  |  |  |  |
| Source of Variation | Df | Sum-of-squares | Mean square | F |
| Column Factor | 7 | 12870 | 1839 | 60.64 |
| Row Factor | 3 | 1445 | 481.8 | 15.89 |
| Residual | 21 | 636.8 | 30.32 |  |
|  |  |  |  |  |
| Number of missing values | 0 |  |  |  |
|  |  |  |  |  |
| Bonferroni posttests |  |  |  |  |
|  |  |  |  |  |
| Ascorbic vs HLE |  |  |  |  |
| Row Factor | Ascorbic | HLE | Difference | 95% CI of diff. |
| 200 | 93.00 | 65.00 | -28.00 | -55.84 to -0.1586 |
| 100 | 89.00 | 57.90 | -31.10 | -58.94 to -3.259 |
| 50 | 84.00 | 52.80 | -31.20 | -59.04 to -3.359 |
| 25 | 78.00 | 51.70 | -26.30 | -54.14 to 1.541 |
|  |  |  |  |  |
| Row Factor | Difference | t | P value | Summary |
| 200 | -28.00 | 3.595 | P<0.01 | ** |
| 100 | -31.10 | 3.994 | P<0.01 | ** |
| 50 | -31.20 | 4.006 | P<0.01 | ** |
| 25 | -26.30 | 3.377 | P < 0.05 | * |
|  |  |  |  |  |
| Ascorbic vs EALE |  |  |  |  |
| Row Factor | Ascorbic | EALE | Difference | 95% CI of diff. |
| 200 | 93.00 | 68.30 | -24.70 | -52.54 to 3.141 |
| 100 | 89.00 | 53.20 | -35.80 | -63.64 to -7.959 |
| 50 | 84.00 | 50.70 | -33.30 | -61.14 to -5.459 |
| 25 | 78.00 | 48.50 | -29.50 | -57.34 to -1.659 |
|  |  |  |  |  |
| Row Factor | Difference | t | P value | Summary |
| 200 | -24.70 | 3.172 | P < 0.05 | * |
| 100 | -35.80 | 4.597 | P<0.001 | *** |
| 50 | -33.30 | 4.276 | P<0.01 | ** |
| 25 | -29.50 | 3.788 | P<0.01 | ** |
|  |  |  |  |  |
| Ascorbic vs HRE |  |  |  |  |
| Row Factor | Ascorbic | HRE | Difference | 95% CI of diff. |
| 200 | 93.00 | 90.50 | -2.500 | -30.34 to 25.34 |
| 100 | 89.00 | 85.00 | -4.000 | -31.84 to 23.84 |
| 50 | 84.00 | 61.20 | -22.80 | -50.64 to 5.041 |
| 25 | 78.00 | 50.90 | -27.10 | -54.94 to 0.7414 |
|  |  |  |  |  |
| Row Factor | Difference | t | P value | Summary |
| 200 | -2.500 | 0.3210 | P > 0.05 | ns |
| 100 | -4.000 | 0.5136 | P > 0.05 | ns |
| 50 | -22.80 | 2.928 | P < 0.05 | * |
| 25 | -27.10 | 3.480 | P<0.01 | ** |
|  |  |  |  |  |
| Ascorbic vs EARE |  |  |  |  |
| Row Factor | Ascorbic | EARE | Difference | 95% CI of diff. |
| 200 | 93.00 | 52.40 | -40.60 | -68.44 to -12.76 |
| 100 | 89.00 | 49.20 | -39.80 | -67.64 to -11.96 |
| 50 | 84.00 | 48.80 | -35.20 | -63.04 to -7.359 |
| 25 | 78.00 | 48.90 | -29.10 | -56.94 to -1.259 |
|  |  |  |  |  |
| Row Factor | Difference | t | P value | Summary |
| 200 | -40.60 | 5.213 | P<0.001 | *** |
| 100 | -39.80 | 5.111 | P<0.001 | *** |
| 50 | -35.20 | 4.520 | P<0.001 | *** |
| 25 | -29.10 | 3.737 | P<0.01 | ** |
|  |  |  |  |  |
| Ascorbic vs 1 |  |  |  |  |
| Row Factor | Ascorbic | 1 | Difference | 95% CI of diff. |
| 200 | 93.00 | 32.30 | -60.70 | -88.54 to -32.86 |
| 100 | 89.00 | 28.50 | -60.50 | -88.34 to -32.66 |
| 50 | 84.00 | 24.00 | -60.00 | -87.84 to -32.16 |
| 25 | 78.00 | 19.20 | -58.80 | -86.64 to -30.96 |
|  |  |  |  |  |
| Row Factor | Difference | t | P value | Summary |
| 200 | -60.70 | 7.794 | P<0.001 | *** |
| 100 | -60.50 | 7.769 | P<0.001 | *** |
| 50 | -60.00 | 7.705 | P<0.001 | *** |
| 25 | -58.80 | 7.550 | P<0.001 | *** |
|  |  |  |  |  |
| Ascorbic vs 3 |  |  |  |  |
| Row Factor | Ascorbic | 3 | Difference | 95% CI of diff. |
| 200 | 93.00 | 39.20 | -53.80 | -81.64 to -25.96 |
| 100 | 89.00 | 35.80 | -53.20 | -81.04 to -25.36 |
| 50 | 84.00 | 28.20 | -55.80 | -83.64 to -27.96 |
| 25 | 78.00 | 16.80 | -61.20 | -89.04 to -33.36 |
|  |  |  |  |  |
| Row Factor | Difference | t | P value | Summary |
| 200 | -53.80 | 6.908 | P<0.001 | *** |
| 100 | -53.20 | 6.831 | P<0.001 | *** |
| 50 | -55.80 | 7.165 | P<0.001 | *** |
| 25 | -61.20 | 7.859 | P<0.001 | *** |
|  |  |  |  |  |
| Ascorbic vs 4 |  |  |  |  |
| Row Factor | Ascorbic | 4 | Difference | 95% CI of diff. |
| 200 | 93.00 | 37.90 | -55.10 | -82.94 to -27.26 |
| 100 | 89.00 | 32.70 | -56.30 | -84.14 to -28.46 |
| 50 | 84.00 | 26.90 | -57.10 | -84.94 to -29.26 |
| 25 | 78.00 | 22.80 | -55.20 | -83.04 to -27.36 |
|  |  |  |  |  |
| Row Factor | Difference | t | P value | Summary |
| 200 | -55.10 | 7.075 | P<0.001 | *** |
| 100 | -56.30 | 7.229 | P<0.001 | *** |
| 50 | -57.10 | 7.332 | P<0.001 | *** |
| 25 | -55.20 | 7.088 | P<0.001 | *** |
